# Supplementary material for: The interactome of intact mitochondria by cross-linking mass spectrometry provides evidence for coexisting respiratory supercomplexes
Source: Mol Cell Proteomics. 2017 Dec 8;17(2):216–32. doi: 10.1074/mcp.RA117.000470 (PMC5795388; doi:10.1074/mcp.RA117.000470)
Supplement: Supplemental Data [file supp_RA117.000470_133922_0_supp_23978_fzffwf.zip › spectra_annotation/mito_DR_spectra_annotation/496-1-7-1-5-1.pdf]

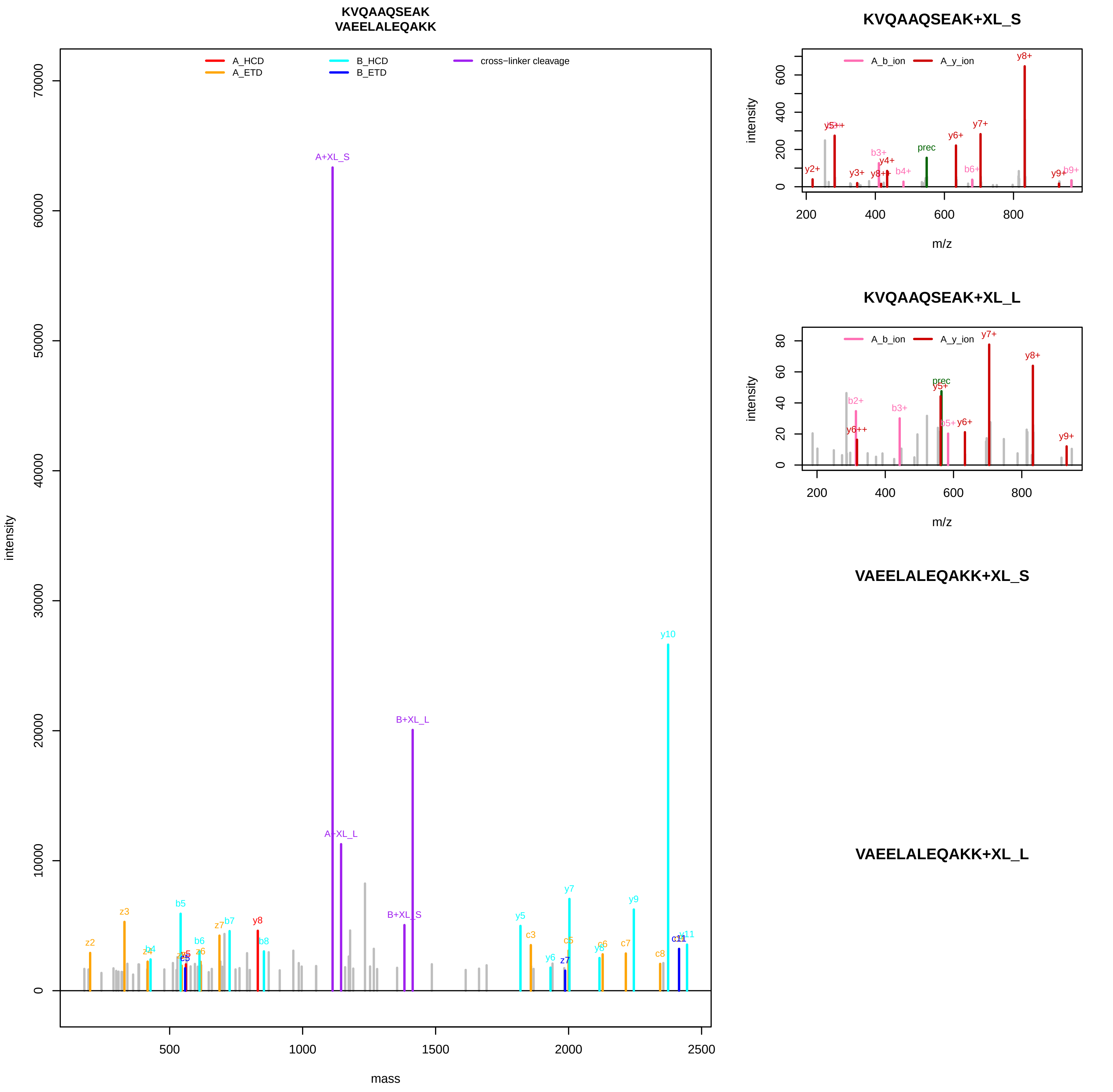
KVQAAQSEAK
VAEELALEQAKK
A_HCD
A_ETD
B_HCD
B_ETD
cross−linker cleavage
intensity
mass
A+XL_S
B+XL_L
A+XL_L
B+XL_S
y10
y7
y9
y5
y8
b5
z3
z2
z7
b7
b6
b8
c3
c5
c7
c8
c11
y11
y6
z7
c6
KVQAAQSEAK+XL_S
A_b_ion
A_y_ion
y8+
y7+
y6+
prec
y5++
y2+
y3+
b3+
y4+
y8++
b4+
b6+
y9+
b9+
m/z
KVQAAQSEAK+XL_L
y5+
b2+
y6++
b5+
VAEELALEQAKK+XL_S
VAEELALEQAKK+XL_L
